# A phase II study of S-1 with concurrent radiotherapy in elderly patients with esophageal cancer

**DOI:** 10.18632/oncotarget.20938

**Published:** 2017-09-15

**Authors:** Yongling Ji, Xianghui Du, Ye Tian, Liming Sheng, Lei Cheng, Ying Chen, Guoqing Qiu, Xia Zhou, Wuan Bao, Danhong Zhang, Ming Chen

**Affiliations:** ^1^ Department of Radiotherapy and Oncology, The Second Affiliated Hospital of Soochow University, 215000 Suzhou, China; ^2^ Department of Radiation Oncology, Zhejiang Cancer Hospital, 310022 Hangzhou, China; ^3^ Zhejiang Key Laboratory of Radiation Oncology, Hangzhou 310022, China

**Keywords:** S-1, chemoradiotherapy, esophageal cancer, elderly

## Abstract

**Background:**

Concurrent chemoradiotherapy (CCRT) using conventional platinum-based doublets are often associated with significant incidence of toxic effects in elderly patients with esophageal cancer. We previously reported a phase I trial of CCRT using S-1, an oral 5-fluorouracil derivative, which yielded well safe and active outcomes.

**Methods:**

Patients with histologically confirmed esophageal cancer, who were age of 70 years or older with performance status (PS) score of 0-2 or age of 66 to 69 with PS score of 2, were eligible for this Phase II trial. Radiotherapy was delivered in 1.8 Gy per fraction to a total dose of 54 Gy. Concurrently, S-1 was administered at 70 mg/m^2^ on days 1–14 and 29–42. The primary end point was 2-year overall survival rate.

**Results:**

Thirty patients were enrolled, and 28 patients completed the full course of radiotherapy. No grade 4 toxicity or treatment-related death occurred. The grade 3 toxicities included esophagitis (16.7%), leucopoenia (13.3%), neutropenia (10%), anaemia (3.3%), pneumonitis (3.3%) and fatigue (3.3%). The median progression-free survival time and median survival time was 19 and 24 months, respectively. The 2-year overall survival rate was 45.1%, which exceeded the predefined threshold of 2-year OS 35% and met the primary end point of the study.

**Conclusions:**

The results suggest that CCRT using S-1 is effective with mild toxicity in elderly patients with esophageal cancer. A phase III trial is needed to further evaluate this regimen.

## INTRODUCTION

Esophageal cancer is a major public health problem in China. The estimated number of new esophageal cancer cases and deaths in 2011 were 291,238 and 218,957, respectively [1, 2]. Esophageal cancer often occurs in elderly patients. Approximately 30-40% of the patients with esophageal cancer were 70 years old or above [3]. Because of the rapid aging population and greater life expectancy, the number of elderly patients in China is likely to increase significantly in the future.

Based on the results of phase III trial RTOG 8501, concurrent chemoradiotherapy (CCRT) using 5-FU and cisplatin has become the standard nonsurgical treatment for patients with localized esophageal cancer[4, 5]. However, 64% of patients treated with CCRT experienced severe or life threatening adverse events, and only 23% of patients enrolled in this study were over age 70, which brought a question about the suitability of CRT for elderly patients. Some retrospective studies suggested that elderly patients might also benefit from CCRT with 5-FU and CDDP, with median survival time of 8.6-15.2 months. However, the toxicity was substantial, and only 9-38.5% of elderly patients could complete the scheduled treatment [6–9]. Therefore, potent new CCRT regimens with lower toxicity need to be investigated for elderly patients with esophageal cancer.

S-1 is an oral 5-fluorouracil derivative agent designed to enhance anticancer activity and to reduce toxicity. We have completed a phase I trial of S-1 with concurrent radiotherapy in elderly patients with esophageal cancer, and found the regimen to be feasible and tolerated [10]. Esophagitis was the most common toxicity in the study, with grade 3 esophagitis observed in 3 of 12 patients. No grade 4 toxicity or treatment-related deaths was observed. The median survival time was 29 months. Against this background, we subsequently conducted a phase II trial of this regimen. The primary end-point was overall survival, and the secondary end-points included toxicities, response rate and progression-free survival.

## RESULTS

### Patient characteristics

From October 2012 to October 2015, 30 patients were enrolled in the study. There were 11 patients aged 66 to 69 with ECOG PS of 2, and 19 patients aged 70 years or older with ECOG PS of 0-2. Patient characteristics are summarized in Table 1. The majority of patients had stage III -IVB disease (80.0%).

**Table 1:** Patient characteristics

Characteristic	No. of Patients (N=30)	Valid Patients (%)
Age(y)		
Median (range)	72(65-80)	
Sex		
Male	23	76.7
Female	7	23.3
ECOG performance status score		
0	4	13.3
1	12	40.0
2	14	46.7
Histology		
Squamous cell carcinoma	29	96.7
Adenocarcinoma	1	3.3
Tumour locations		
Cervical	2	6.7
Upper third	11	36.7
Middle third	12	40.0
Lower third	5	16.7
Tumour length		
<5 cm	5	16.7
≥5–<10	21	70.0
≥10	4	13.3
T Stage		
T1	1	3.3
T2	5	16.7
T3	14	46.7
T4	10	33.3
N Stage		
N0	7	23.3
N1	23	76.7
Clinical Stage		
I	1	3.3
IIa	2	6.7
IIb	3	10.0
III	21	70.0
IVa	1	3.3
IVb	2	6.7
Histologic grade^a^		
Gx	11	36.7
G1	0	0
G2	12	40.0
G3	6	20.0
G4	1	3.3
Weight loss in 6 months		
<10%	17	56.7
≥10%	13	43.3
(Range)	1(0-3)	

^a^, Gx, grade could not be assessed; G1, well differentiated; G2, moderately differentiated; G3, poorly differentiated; G4, undifferentiated.

### Treatment delivery

Of the 30 patients enrolled in the study, 28 patients completed the full course of radiotherapy. One patient with PS 2 refused to continue at 28.8 Gy because of grade 3 fatigue. Another patient refused to continue at 45Gy because of grade 3 esophagitis. Full dose chemotherapy was completed in 16 patients (53.3%). The mean proportion of the actual S-1 dose delivered was 89% (range, 28.6%–100%). The reasons for dose reduction of S-1 were adverse events (n=12), repeatedly symptomatic atrial fibrillation (n=1), and angina (n=1).

### Toxicity

All 30 patients received a toxicity evaluation. The toxicities occurred are listed in Table 2. The grade 3 toxicities included esophagitis (16.7%), leucopoenia (13.3%), neutropenia (10%), anaemia (3.3%), pneumonitis (3.3%) and fatigue (3.3%). No grade 4 toxicity or treatment-related death occurred.

**Table 2:** Adverse events

	Grade 1		Grade 2		Grade 3		Grade 4	
Toxicity	No.	%	No.	%	No.	%	No.	%
Leucopoenia	9	30	15	50	4	13.3	0	0
Neutropenia	16	53.3	9	30	3	10	0	0
Anaemia	18	60	6	20	1	3.3	0	0
Thrombocytopenia	14	46.7	3	10	0	0	0	0
Esophagitis	15	50	6	20	5	16.7	0	0
Pneumonitis	2	6.7	2	6.7	1	3.3	0	0
Nausea/vomiting	3	10	1	3.3	0	0	0	0
Diarrhoea	1	3.3	0	0	0	0	0	0
Fatigue	8	26.7	2	6.7	1	3.3	0	0
Anorexia	3	10	2	6.7	0	0	0	0

### Efficacy and survival

A total of 29 patients were eligible for response evaluation. Six and 11 patients experienced complete responses (20%) and partial responses (36.7%), respectively. Twelve patients exhibited stable disease (40%). One patient who discontinued radiotherapy at 28.8Gy did not receive response evaluation.

**Figure 1:**
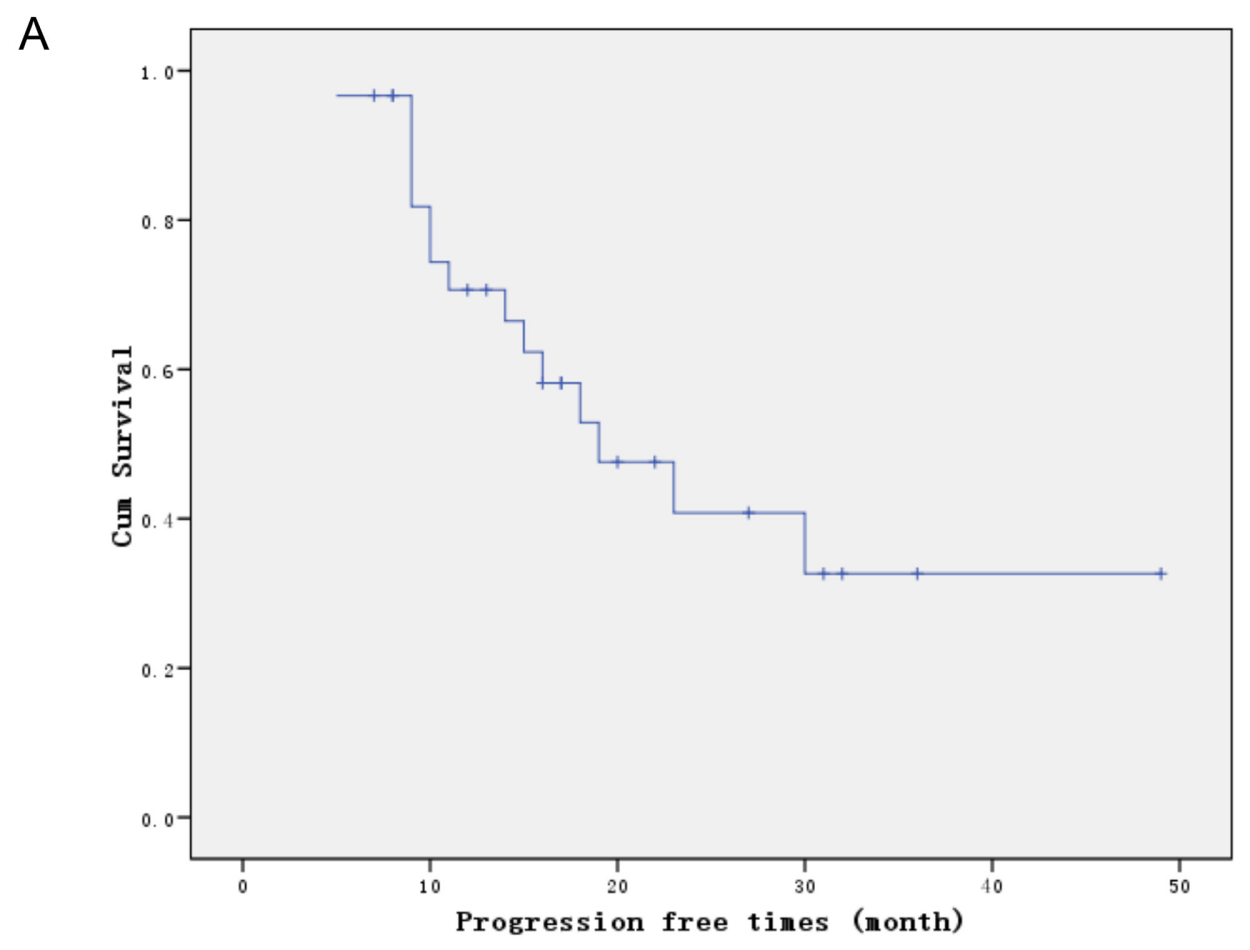

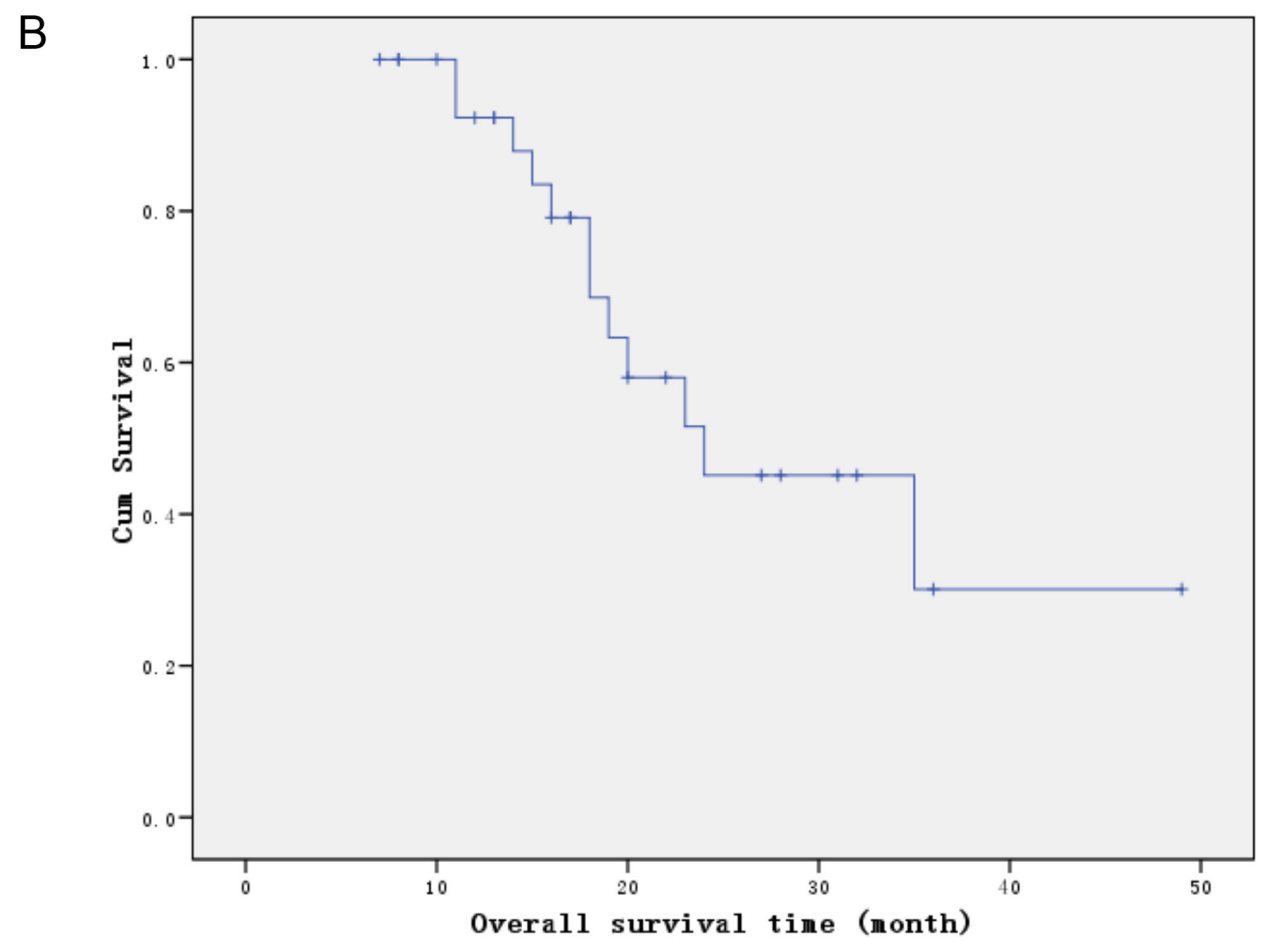
Survival curves **(A)** Progression free survival. **(B)** Overall survival.

After a median follow-up period of 18 months (range, 10–41.0 months), 15 patients had experienced recurrences, of which 7 (46.6%) were locoregional, 6 (40.0%) distant and 2 (13.3%) both locoregional and distant. The median progression-free survival time was19.0 months and the 2-year progression-free survival rate was 40.8% (Figure 1). The median survival time and the 2-year overall survival rate were 24 months and 45.1% (Figure 1), respectively. There was no significant difference in 2-year OS between stage I-II and III-IV (83.3% vs 40.5%, P=0.268), T1-3 and T4 (48.3% vs 35.0%,P=0.308), N0 and N1 (55.6% vs 43.9%,P=0.868), age 65-69 and ≥ 70 (61.0% vs 33.9%, p=0.286), PS 0-1 and 2 (46.3% vs 44.4%, p=0.952). No prognostic factor was identified in multivariate analyses.

## DISCUSSION

In the current phase II trial, we assessed the toxicity and efficacy of CCRT using S-1 in the treatment of esophageal cancer in elderly patients. In addition to patients aged 70 years or older, this study also enrolled patient aged 65-69 with poor performance status (PS 2), who was considered to be unable to tolerate CCRT using conventional platinum-based doublets. Our results suggest that this CCRT regimen is safe and efficacious in this population.

The median survival time in the present study was 24 months, and the 2-year OS was 45.1%, which exceeded the predefined threshold of 2-year OS 35% and met the primary end point of the study. These results were better than previous reports in CCRT using 5-FU plus platinum in elderly patients, with median survival time of 8.6-15.2 months, and 2-year OS of 34.5-35.5% [6, 7, 11]. In the largest study on elderly patients, Tougeron et al reported 109 patients received CCRT using 5-FU and CDDP (n=98) or CPT-11 and CDDP, 47.7% of patients had stage III -IVB disease, 79.8% of patients had good performance status (PS 0-1). The median survival was 15.2 months, and the 2-year survival was 35.5% [6]. Recently, Song et al retrospective analyzed the efficacy of CCRT using PTX and CDDP in patients aged 70 years or older. The 2-year OS for stage I–II and III–IV were 76.0%, and 38.6%, respectively [12]. In our study, the 2-year OS for stage I–II and III–IV were 83.3%, and 40.5%, respectively. The good survival results in our study might be attributable partly to the good radiosensitizing effect of S-1. Studies have shown that the half-life of plasma concentrations of 5-Fu after oral S-1 was significantly prolonged compared with that of 5-Fu after intravenous administration, and prolonged exposure is desirable in order to achieve radiosensitisation [13]. Furthermore, Gimeracil, a component of S-1, has been found to enhance the efficacy of RT through the inhibition of the repair of radiation-induced DNA damage [14, 15]. In a preclinical study using human cancer xenograft models, oral S-1 produced better response than intravenous 5-FU in the CCRT [15].

Several studies in non-age-selected patients also showed CCRT using S-1 and platinum had very promising efficacy for esophageal cancer. In a prospective study of CCRT with S-1 and nedaplatin in 20 patients with stage II/III esophageal cancer (median age 65, range 50-75), the 3-year overall survival was 58.0% [16]. In a phase II trial of preoperative CCRT using S-1 and CDDP in patients with stage IIA-IVA esophageal cancer, twenty-five patients underwent esophagectomy following chemoradiation, and 15 achieved complete pathologic regression. The 2-years overall survival was 65 % [17]. In another phase II study of 116 patients received CCRT with S-1 and CDDP, Iwase et al [18] reported the median survival time was 7.0, 2.6, and 1.3 years for the stage II, III, and IVa patients, respectively [18]. These results seemed to be better than that of CCRT using 5-FU and CDDP, with a median survival time of 13-18 months [4, 5, 19, 20].

Elderly patients were at increased risk for treatment toxicity, because of the reduced physiologic reserves in all organs, especially the bone marrow reserve and the renal function. Furthermore, comorbidities were highly prevalent in elderly patients, which may also decrease the tolerance to CCRT. In a retrospectively analysis of CCRT using 5-FU and CDDP in 33 elderly patients and 145 non-elderly patients with esophageal cancer, the incidences of over grade 3 leukopaenia was 70% versus 49.7%, anaemia 51.50% versus 17.9%, and thrombocytopaenia 33.3% versus 18%, respectively [7]. And only 33.3% of the elderly patients completed the planned treatment, comparing to 68.3% of non-elderly patients [7]. In another retrospective analysis of CCRT using 5-FU and platinum in patients aged over 75, treatment-related death was suspected in up to 18% of patients, grade 4 leukocytopenia and thrombocytopenia occurred in 14% and 18% of patients, even both chemotherapy and radiotherapy were reduced in dose and field where necessary. Therefore, potent new CCRT regimen with lower toxicity needs to be investigated. Our study demonstrated that the toxicity of CCRT using S-1 was mild in elderly patients with esophageal caner. No grade 4 toxicity or treatment-related death occurred. The most common grade 3 toxicities were esophagitis (16.7%), leucopoenia (13.3%) and neutropenia (10%). Grade 3 pneumonitis occurred in 1 patient (3.3%). Similarly, a phase II study of elderly patients with locally advanced non-small-cell lung cancer also showed CCRT using S-1 had mild toxicity. The most common grade 3 toxicities were neutropenia and pneumonitis, which were observed in 17% and 10% of patients, respectively. And also, no grade 4 toxicity or treatment-related death occurred in this study [21].

In a series of studies on CCRT using 5-FU and platinum in non-age-selected patients with esophageal cancer, two cycles of consolidation chemotherapy were given after the completion of CCRT [4, 20]. However, the consolidation chemotherapy were not used in our study, because of the following considerations: Firstly, the compliance of consolidation chemotherapy in elderly patients is very poor. Previous studies reported that 61.5-91% of elderly patients couldn’t complete the scheduled consolidation chemotherapy with 5-FU and platinum [6–9]. Secondly, there is no evidence to support any benefit of the consolidation chemotherapy. In a retrospective analysis for patients aged over 75, Wakui et al found that the consolidation chemotherapy of 5-FU and platinum had a minor effect on DFS. They suggested that elderly patients do not have to receive the consolidation chemotherapy after CCRT. At last, many patients in our study who come from rural areas were lack of good care. The consolidation chemotherapy at home may increase the risk of complications, such as infections, malnutrition or falls, especially for the elderly patients with poor PS or comorbidities. Servagi-Vernat et al also conducted a phase II study of CCRT without consolidation chemotherapy in 22 elderly patients with esophageal cancer. The regimen consisted of radiotherapy 50Gy concurrent with CDDP 75 mg/m^2^ on days 1 and 21. All patients completed the planned treatment, with no grade 4 toxicity observed. A total of 63.6% patients achieved CR, and the median survival time was 15 months [22].

In conclusion, the administration of concurrent chemoradiotherapy with S-1 demonstrated favorable efficacy, with acceptable toxicity, in elderly patients with esophageal cancer. We have already started a multicenter, randomized, phase III trial to further evaluate the efficacy of this regimen.

## MATERIALS AND METHODS

### Eligibility criteria

Eligible patients were required to meet the following criteria: 1) histologically confirmed squamous cell carcinoma or adenocarcinoma; 2) age ≥ 70 years old with Eastern Cooperative Oncology Group (ECOG) PS score of 0-2; or age 65-69 years old with PS score of 2; 3) stage I to IV diseases according to the 2002 (version 6.0) American Joint Committee on Cancer staging system, with the exception of stage IVb of distant and hematogenous visceral metastasis (eligible if it was lymph node metastases); 4) 12 weeks or more life expectancy; 5) adequate bone marrow reserve (leukocyte count ≥ 4,000 mm^3^, neutrophil count ≥ 2,000 mm^3^, platelet count ≥ 100,000 mm^3^, and haemoglobin ≥ 10 g/dL); 6) normal liver function (total serum bilirubin ≤ 1.5 mg/dL, with aspartate transaminase and alanine transaminase levels being lower than double of the upper normal limit); 7) normal renal function (normal serum creatinine and blood urea nitrogen levels); and 8) adequate pulmonary function (FEV1>1 L). Patients were excluded if they had one of the following: 1) malignant pleural or pericardial effusion; 2) a concomitant serious illness such as uncontrolled angina pectoris; 3) myocardial infarction in previous 3 months; 4) heart failure; 5) interstitial pneumonia; or 6) infection or other diseases contraindicating chemotherapy or RT. The study was approved by the Ethics Committee of Zhejiang Cancer Hospital, and the written informed consent was obtained from all patients.

### Pre-treatment evaluation

The pre-treatment evaluation included history, physical examination, electrocardiography, and assessment of bone marrow, renal, and hepatic functions. The items of the disease evaluation included a neck, chest and abdominal CT, an upper GI endoscopy, endoscopic ultrasound (EUS), and barium esophagraphy. Bronchoscopy was performed for cervical or mid-esophageal tumours. A brain MRI, and radionuclide bone scan were performed if clinically indicated. PET-CT scan was recommended but not a requisite part of the pretreatment evaluation. The clinical TNM system stage was determined according to the 2002 (version 6.0) American Joint Committee on Cancer staging system. The Charlson score was used for the analysis of patient comorbidity [23].

### Treatment

Patients received an oral dose of S-1 70 mg/m^2^ per day on days 1–14 and 29–42. Because S-1 is only available for use in 20mg capsules in our hospital, the individual dose was rounded down to the nearest pill size less than the calculated dose. A powder form of S-1 would be administered if patients could not swallow the oral capsule.

RT was administered beginning on day 1 of chemotherapy using a linear accelerator (10 MeV). Because that the chemotherapy intensity in our trial (s-1 only) was lower than that of RTOG 8501 and INT 0123 (5-FU + cisplatin), higher dose of RT was given. Each patient received a single 1.8Gy daily fraction for 5 consecutive days each week, until a total dose of 54 Gy was reached. Each patient underwent a treatment-planning CT scan with intravenous contrast. The gross tumour volume (GTV) was contoured based on the EUS, barium esophagraphy and chest CT scans. The clinical target volume (CTV) consisted of the GTV plus a 0.5-1cm circumferential margin and 3-4cm cranio-caudal margin. The supraclavicular nodes were included for upper esophageal lesions, and celiac nodes were included for distal esophageal lesions. The planning target volume (PTV) consisted of the CTV plus a 0.5–1cm margin for daily set-up error and organ motion. Dose-volume histogram analysis was required to ensure that the spinal cord, lung, heart, and liver exposure were within organ tolerance.

Dose and schedule modifications were identical to those of the phase I trial [10].If Grade 3 neutropenia alone occurred, S-1 was held and RT continued. S-1 was then restarted at the same dose when the absolute neutrophil count became ≥1000/mm3. If other Grade 3 or 4 toxicities occurred, both RT and S-1 were held until recovery to grade 2.

### Assessment of response and toxicity

Clinical response was assessed according to RECIST (Response Evaluation Criteria in Solid Tumours)[24]. This evaluation was performed 4–6 weeks after CCRT completion. Endoscopy and CT scans were performed every 3 months during follow-up.

Toxicity was assessed based on the National Cancer Institute Common Terminology Criteria for Adverse Events v3.0. A complete blood cell count and serum chemistry profile was performed at least once a week during treatment. Non-hematologic toxicity was evaluated on a daily basis via interview and physical examination throughout the treatment period.

### Statistical analysis

The primary objective of this study was 2-year survival rate. The secondary end-points included toxicities, response rate and progression-free survival. It was estimated based on previous reports that the 2-year OS rate after radiotherapy alone was about 15%[8]. To detect an improvement in the 2-year OS rate from 15% to 35% with a significance level of 0.05 and 80% power, and if the dropout rate was approximately 10%, a total of 30 patients were required. The survivals time was calculated from the date of registration to the first documented date of disease progression, or the date of death, respectively, using the Kaplan–Meier method. All p values were calculated in a two-tailed manner, and the significance level was set at p < 0.05. Statistical analyses were conducted using SPSS software (Version 20; SPSS Inc., Chicago, IL, USA).

## References

[1] Zeng H, Zheng R, Zhang S, Zuo T, Xia C, Zou X, Chen W (2016). Esophageal cancer statistics in china, 2011: estimates based on 177 cancer registries. Thorac Cancer.

[2] Chen W, Zheng R, Baade PD, Zhang S, Zeng H, Bray F, Jemal A, Yu XQ, He J (2016). Cancer statistics in China, 2015. CA Cancer J Clin.

[3] Wu M, Van't Veer P, Zhang ZF, Wang XS, Gu XP, Han RQ, Yang J, Zhang XF, Liu AM, Kok FJ, Kampman E, Zhao JK (2011). A large proportion of esophageal cancer cases and the incidence difference between regions are attributable to lifestyle risk factors in China. Cancer Lett.

[4] Cooper JS, Guo MD, Herskovic A, Macdonald JS, Martenson JA, Al-Sarraf M, Byhardt R, Russell AH, Beitler JJ, Spencer S, Asbell SO, Graham MV, Leichman LL (1999). Chemoradiotherapy of locally advanced esophageal cancer: long-term follow-up of a prospective randomized trial (RTOG 85-01). JAMA.

[5] Herskovic A, Martz K, al-Sarraf M, Leichman L, Brindle J, Vaitkevicius V, Cooper J, Byhardt R, Davis L, Emami B (1992). Combined chemotherapy and radiotherapy compared with radiotherapy alone in patients with cancer of the esophagus. N Engl J Med.

[6] Tougeron D, Di Fiore F, Thureau S, Berbera N, Iwanicki-Caron I, Hamidou H, Paillot B, Michel P (2008). Safety and outcome of definitive chemoradiotherapy in elderly patients with oesophageal cancer. Br J Cancer.

[7] Takeuchi S, Ohtsu A, Doi T, Kojima T, Minashi K, Mera K, Yano T, Tahara M, Muto M, Nihei K (2007). A retrospective study of definitive chemoradiotherapy for elderly patients with esophageal cancer. Am J Clin Oncol.

[8] Semrau R, Herzog SL, Vallbohmer D, Kocher M, Holscher A, Muller RP (2012). Radiotherapy in elderly patients with inoperable esophageal cancer. is there a benefit?. Strahlenther Onkol.

[9] Wakui R, Yamashita H, Okuma K, Kobayashi S, Shiraishi K, Terahara A, Sasano N, Ohtomo K, Nakagawa K (2010). Esophageal cancer: definitive chemoradiotherapy for elderly patients. Dis Esophagus.

[10] Ji Y, Qiu G, Sheng L, Sun X, Zheng Y, Chen M, Du X (2016). A phase I dose escalation study of S-1 with concurrent radiotherapy in elderly patients with esophageal cancer. J Thorac Dis.

[11] Rochigneux P, Resbeut M, Rousseau F, Bories E, Raoul JL, Poizat F, Moureau-Zabotto L (2014). Radio(chemo)therapy in elderly patients with esophageal cancer: a feasible treatment with an outcome consistent with younger patients. Front Oncol.

[12] Song T, Zhang X, Fang M, Wu S (2015). Concurrent chemoradiotherapy using paclitaxel plus cisplatin in the treatment of elderly patients with esophageal cancer. Onco Targets Ther.

[13] van Groeningen CJ, Peters GJ, Schornagel JH, Gall H, Noordhuis P, de Vries MJ, Turner SL, Swart MS, Pinedo HM, Hanauske AR, Giaccone G (2000). Phase I clinical and pharmacokinetic study of oral S-1 in patients with advanced solid tumors. J Clin Oncol.

[14] Takagi M, Sakata K, Someya M, Tauchi H, Iijima K, Matsumoto Y, Torigoe T, Takahashi A, Hareyama M, Fukushima M (2010). Gimeracil sensitizes cells to radiation via inhibition of homologous recombination. Radiother Oncol.

[15] Fukushima M, Sakamoto K, Sakata M, Nakagawa F, Saito H, Sakata Y (2010). Gimeracil, a component of S-1, may enhance the antitumor activity of X-ray irradiation in human cancer xenograft models *in vivo*. Oncol Rep.

[16] Tsuda T, Inaba H, Miyazaki A, Izawa N, Hirakawa M, Watanabe Y, Kitajima S, Hoshikawa Y, Gomi H, Kimura M, Itoh F (2011). Prospective study of definitive chemoradiotherapy with S-1 and nedaplatin in patients with stage II/III (non-T4) esophageal cancer. Esophagus.

[17] Chang H, Shin SK, Cho BC, Lee CG, Kim CB, Kim DJ, Lee JG, Hur J, Lee CY, Bae MK, Kim HR, Lee SK, Park JC (2014). A prospective phase II trial of S-1 and cisplatin-based chemoradiotherapy for locoregionally advanced esophageal cancer. Cancer Chemother Pharmacol.

[18] Iwase H, Shimada M, Tsuzuki T, Hirashima N, Okeya M, Hibino Y, Ryuge N, Yokoi M, Kida Y, Kuno T, Tanaka Y, Kato B, Esaki M (2013). Concurrent chemoradiotherapy with a novel fluoropyrimidine, S-1, and cisplatin for locally advanced esophageal cancer: long-term results of a phase II trial. Oncology.

[19] Minsky BD, Pajak TF, Ginsberg RJ, Pisansky TM, Martenson J, Komaki R, Okawara G, Rosenthal SA, Kelsen DP (2002). INT 0123 (Radiation Therapy Oncology Group 94-05) phase III trial of combined-modality therapy for esophageal cancer: high-dose versus standard-dose radiation therapy. J Clin Oncol.

[20] Conroy T, Galais MP, Raoul JL, Bouche O, Gourgou-Bourgade S, Douillard JY, Etienne PL, Boige V, Martel-Lafay I, Michel P, Llacer-Moscardo C, Francois E, Crehange G (2014). Definitive chemoradiotherapy with FOLFOX versus fluorouracil and cisplatin in patients with oesophageal cancer (PRODIGE5/ACCORD17): final results of a randomised, phase 2/3 trial. Lancet Oncol.

[21] Aoe K, Takigawa N, Hotta K, Maeda T, Kishino D, Nogami N, Tabata M, Harita S, Okada T, Kubo T, Hosokawa S, Fujiwara K, Gemba K (2014). A phase II study of S-1 chemotherapy with concurrent thoracic radiotherapy in elderly patients with locally advanced non-small-cell lung cancer: the Okayama Lung Cancer Study Group Trial 0801. Eur J Cancer.

[22] Servagi-Vernat S, Bosset M, Crehange G, Buffet-Miny J, Puyraveau M, Maingon P, Mercier M, Bosset JF (2009). Feasibility of chemoradiotherapy for oesophageal cancer in elderly patients aged >or=75 years: a prospective, single-arm phase II study. Drugs Aging.

[23] Charlson ME, Pompei P, Ales KL, MacKenzie CR (1987). A new method of classifying prognostic comorbidity in longitudinal studies: development and validation. J Chronic Dis.

[24] Therasse P, Arbuck SG, Eisenhauer EA, Wanders J, Kaplan RS, Rubinstein L, Verweij J, Van Glabbeke M, van Oosterom AT, Christian MC, Gwyther SG (2000). New guidelines to evaluate the response to treatment in solid tumors. European Organization for Research and Treatment of Cancer, National Cancer Institute of the United States, National Cancer Institute of Canada. J Natl Cancer Inst.

